# *Lysinibacillus sphaericus* exposure impedes *Anopheles dirus*’s oviposition via downregulating *vitellogenin*

**DOI:** 10.1186/s13071-025-06745-8

**Published:** 2025-03-21

**Authors:** Shasha Yu, Zhilong Liu, Jing Wang, Hong Zheng, Shiqian Han, Feifei Zheng, Dan Zheng, Caizhi Zhao, Xin Li, Tingting Liu, Xuesen Yang, Ying Wang

**Affiliations:** 1 Department of Tropical Medicine, College of Military Preventive Medicine, Army Medical University (Third Military Medical University), No.30 Gaotanyan Street, Shapingba District, Chongqing, 400038 China; 2 Department of Thoracic Surgery, Xinqiao Hospital, Army Medical University (Third Military Medical University), Chongqing, 400037 China; 3 Frontier Medical Training Brigade, Army Medical University (Third Military Medical University), Hutubi, 831200 Xinjiang China

**Keywords:** *Lysinibacillussphaericus*, *Anopheles dirus*, Fecundity, Vitellogenin, Target of rapamycin pathway

## Abstract

**Background:**

Vector control using *Lysinibacillus sphaericus* is an effective strategy for preventing the transmission of mosquito-borne diseases. Our previous study demonstrated that exposure to *L. sphaericus* during the larval stage of *Anopheles dirus* significantly reduced the fecundity of surviving adult mosquitoes. However, the underlying mechanisms driving this reduction remain unclear. Sublethal doses of *L. sphaericus*, often resulting from insufficient or delayed application, can still impact mosquito populations. Therefore, this study aimed to investigate how sublethal doses of *L. sphaericus* inhibit the reproductive capacity of *An. dirus* mosquitoes.

**Methods:**

First, the staining method was used to detect *L. sphaericus* in surviving adult mosquitoes that had been exposed to sublethal doses during the larval stage. Second, adult mosquitoes were fed a sucrose solution containing *L. sphaericus*, and the effects on the reproductive capacity were observed. Third, transcriptome sequencing and qPCR were employed to identify and validate genes associated with oviposition suppression in *An. dirus* following treatment with sublethal doses of *L. sphaericus*. Finally, we assessed the effects of sublethal doses and direct feeding of *L. sphaericus* on vitellogenin (*Vg*) expression and activation of the target of rapamycin (TOR) signaling pathway using qPCR and Western blotting.

**Results:**

Our findings demonstrated that *L. sphaericus* persists in adult *An*. *dirus* mosquitoes that survived larval exposure to sublethal doses. Additionally, feeding adult female mosquitoes with *L. sphaericus* significantly suppressed their oviposition ability. Transcriptome analysis revealed substantial alterations in gene expression profiles among surviving mosquitoes exposed to sublethal doses of *L. sphaericus*. Notably, *L. sphaericus* inhibit lysosomal function and lipid metabolism, which are critical for mosquito physiology. Furthermore, *L. sphaericus* significantly downregulated the Akt-TOR signaling pathway and *Vg* expression in adult mosquitoes.

**Conclusions:**

Exposure *An. dirus* larvae to *L. sphaericus* resulted in the persistence of *L. sphaericus* in surviving adult mosquitoes and significantly suppressed female oviposition by downregulating *Vg* expression via inhibition of lysosomal function and the TOR signaling pathway. This study offers novel insights into the interaction between *L. sphaericus* and its mosquito host and identifies potential molecular targets for controlling mosquito population density by modulating oviposition behavior.

**Graphical Abstract:**

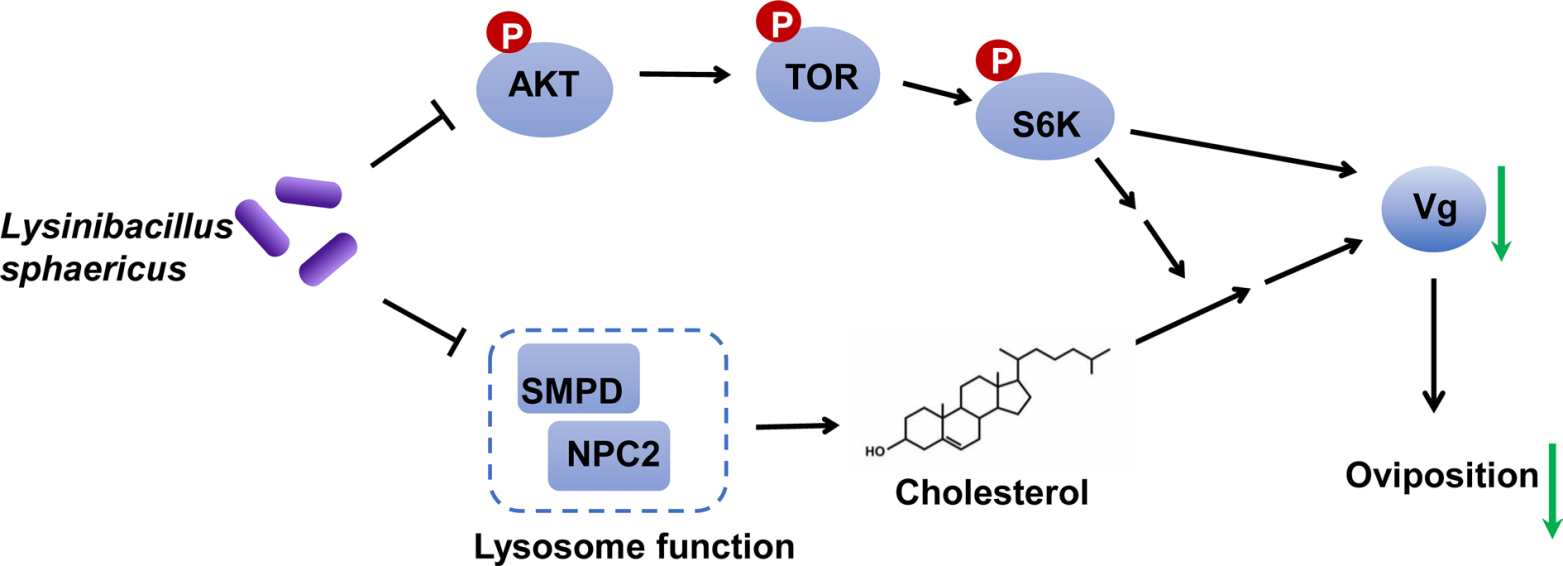

**Supplementary Information:**

The online version contains supplementary material available at 10.1186/s13071-025-06745-8.

## Background

Mosquitoes are major vectors for a variety of infectious diseases, including malaria, dengue fever, West Nile encephalitis, and Zika fever [1]. The World Health Organization’s 2023 report on malaria highlighted that there were 249 million cases of malaria globally, resulting in approximately 608,000 fatalities [2]. Additionally, the incidence and number of dengue fever cases in epidemic regions worldwide have increased rapidly [3]. These mosquito-borne diseases have become increasingly serious global public health concerns. Although treatments for mosquito-borne infections exist, the lack of effective vaccines limits the overall effectiveness of current interventions [4]. Consequently, insecticides use remains a primary strategy for controlling mosquito populations and reducing disease transmission. However, the emergence and spread of mosquito resistance to chemical insecticides [5, 6] have prompted the adoption of biolarvicides as an important alternative strategy for malaria control [7].

*Lysinibacillus sphaericus* is a highly effective and widely used biological insecticide because of its selective toxicity against mosquitoes, safety for non-target organisms, and environmentally friendly degradation profile [8, 9]. Therefore, the use of *L. sphaericus* has become the primary non-chemical method for controlling mosquito populations in Africa and other regions [10, 11]. However, numerous field studies have reported the practical challenges associated with sublethal doses of *L. sphaericus* [12–14]. These challenges are primarily attributed to (i) human factors, such as inaccurate dosage measurement and poorly calibrated weighing instruments; (ii) biological factors, such as vegetation hindering larval contact with *L. sphaericus*; and (iii) abiotic factors, such as water pH, turbidity, and temperature, which can degrade or alter the activity of *L. sphaericus*. These sublethal doses may reduce the efficiency of *L. sphaericus* and alter the physiological characteristics of surviving mosquitoes, thereby affecting mosquito-borne prevention efforts [10].

Our previous studies demonstrated that exposure of *Anopheles dirus* (*An. dirus*) larvae to *L. sphaericus* significantly reduces oviposition and increases egg retention in surviving adult mosquitoes [15]. However, the underlying mechanisms remain unclear. Several studies have shown that larval exposure to bacteria can alter various biological characteristics of adult mosquitoes, including reproduction, lifespan, and body size. For instance, Mahajan et al. [16] observed shortened wing length in male *Culex pipiens quinquefasciatus* and reduced fecundity capacity in larvae continuously exposed to *Francisella tularensis* LVS. Gowelo et al. [17] noted that larval exposure to *Bacillus thuringiensis* in *Anopheles coluzzii* decreased adult lifespan but increased body size, potentially enhancing reproductive capacity through greater blood absorption. Despite these observations, a comprehensive understanding of how larval bacterial exposure affects adult mosquito reproduction remains elusive.

As anautogenous insects, female *Anopheles* mosquitoes rely on vertebrate blood meals to obtain the nutrients required for ovarian development and egg production. Vitellogenesis, a key process in female reproduction, involves the synthesis of vitellogenin (Vg) by fat body cells, its secretion into the hemolymph, and subsequent uptake by oocytes through receptor-mediated endocytosis to nourish developing eggs [18]. The synthesis of Vg is regulated by various factors, with the amino acid-mediated target of rapamycin (TOR) signaling pathway playing a pivotal role [19, 20]. In this study, we investigated the mechanisms underlying the inhibitory effects of sublethal doses of *L. sphaericus* on the reproductive capacity of *An. dirus*. Unveiling these mechanisms will provide a theoretical basis for the application of *L. sphaericus*, predict mosquito population dynamics and disease outbreaks and offer novel molecular targets for reducing mosquito reproductive potential and controlling population density.

## Methods

### Source of *Lysinibacillus sphaericus* 2362, *Anopheles dirus* Hainan strain, and mice

The *Lysinibacillus sphaericus* 2362 strain, in the form of water-dispersible granules with a titer of 1500 IU/mg, was manufactured and supplied by Hubei Kangxin Agricultural Pharmaceutical Company Co., Ltd. The Hainan strain of *Anopheles dirus* was maintained in our laboratory under controlled conditions for an extended period. These conditions included a simulated circadian rhythm (12 h of darkness followed by 12 h of light) at a temperature of 28 °C and relative humidity ranging from 70 to 80%. Larvae were fed a diet of homemade pork liver yeast powder, while adult mosquitoes were provided with a 10% sucrose solution. Kunming mice (weighing 16–20 g) were obtained from the Laboratory Animal Center of the Army Military Medical University. All animal procedures were conducted in accordance with the guidelines approved by the Laboratory Animal Welfare and Ethics Committee of Army Medical university (approval code: AMUWEC20230233).

### Exposure of An. dirus larvae to sublethal doses of L. sphaericus

We followed a similar methodology as previously described [21], where a sublethal dose of the *L. sphaericus* 2362 strain was administered to third-instar larvae for 48–72 h to simulate field conditions with limited use of *L. sphaericus*, based on the bioassay results. Surviving larvae were allowed to develop into pupae, which were then collected and reared to adulthood under conventional conditions identical to those of the untreated group.

### Determination of L. sphaericus persistence in the surviving adult mosquitoes

The persistence of *L. sphaericus* was assessed to determine its intensity after exposure during the larval stage [22]. Twenty surviving female mosquitoes from each group (control, unprimed at the larval stage, and *L. sphaericus* primed at the larval stage) were anesthetized by cold treatment at 4 °C for 10 min, followed by a 30-s immersion in 70% alcohol to eliminate bacteria on the cuticle. After immersion in alcohol, each adult mosquito was rinsed in sterile water to remove excess alcohol and then homogenized with a biovortexer in 50 μl sterile PBS buffer. Subsequently, 20 μl of the resulting macerate was added to 4 ml Luria-Bertani (LB) containing chloramphenicol and streptomycin sulfate (16 μg/μl). Following incubation at 30 ℃ for 12 h with shaking at 200 rpm, 100 μl from each tube was transferred into individual wells of a 96-microwell plate, and turbidity was measured at OD_650_. Turbidity intensity reflects the population density of bacteria in a culture. Meanwhile, spore staining was used to ascertain the spore morphology and confirm the presence of *L. sphaericus*, using a 5% aqueous solution of Malachite Green and a 0.5% aqueous solution of Safranin.

### Assessment of oviposition capacity in female mosquitoes following ingestion of *L. sphaericus* supplemented sucrose solution

Newly emerged adult mosquitoes were fed a sucrose solution containing *L. sphaericus* at concentrations of 0.015 mg/l, 0.03 mg/l, and 0.06 mg/l. The 0.015 mg/l concentration was used for oviposition assays and subsequent Western blot experiments, while all three concentrations were used for quantitative real-time PCR experiments. Mosquitoes were fed with these solutions for 3 consecutive days. Kunming mice were used for blood feeding of *L. sphaericus*-exposed and control mosquitoes. After a 24-h period following the blood meal, engorged female mosquitoes were separated and maintained individually. Oviposition was observed daily for 10 consecutive days. If eggs were laid, the filter paper within each cup was replaced, and the number of eggs was counted under a stereomicroscope.

### Extraction of RNA from *L. sphaericus*-exposed adults of *An. dirus*

Mosquito larvae were treated with sublethal doses of *L. sphaericus*, and surviving adults were collected and fed blood 3 days after eclosion. Twenty-four hours after blood-feeding, engorged female mosquitoes were separated. Total RNA was extracted from 30 female mosquitoes per group using TRIzol®Reagent (Invitrogen, Carlsbad, CA, USA) according to the manufacturer’s instructions. The experimental design included two groups: the *L. sphaericus* exposure group (AdLs) and the control group (AdCtrl), with each group comprising three independent biological replicates. RNA quality was determined using a 5300 Bioanalyzer (Agilent, Santa Clara, CA, USA) and quantified using an ND-2000 (NanoDrop Technologies). Only high-quality RNA samples (OD_260/280_ = 1.8–2.2, OD_260/230_ ≥ 2.0, RIN ≥ 6.5, 28S:18S ≥ 1.0, > 1 μg) were used to the construct sequencing library.

### Library preparation and sequencing

The libraries were prepared, and sequencing procedures were carried out by Shanghai Majorbio Bio Pharm Biotechnology Co., Ltd. (Shanghai, China), following the manufacturer’s guidelines provided by Illumina (San Diego, CA, USA). The mosquito RNA-seq transcriptome library was prepared using 1 μg total RNA in conjunction with Illumina’s Stranded mRNA Prep Ligation kit. Messenger RNA was initially isolated through poly(A) selection using oligo (dT) beads, followed by fragmentation in a fragmentation buffer. Subsequently, double-stranded cDNA synthesis was performed using random hexamer primers supplied by Illumina and the SuperScript double-stranded cDNA synthesis kit from Invitrogen (Carlsbad, CA, USA). Adhering to Illumina's library construction guidelines, the synthesized cDNA underwent end-repair, phosphorylation and ‘A’ base addition. Size selection for 300-bp cDNA target fragments was carried out on a 2% low-range ultra-agarose gel, followed by PCR amplification for 15 cycles using Phusion DNA polymerase (NEB). After quantification with the Qubit 4.0 Fluorometer assay, the paired-end RNA-seq library was sequenced on a NovaSeq 6000 sequencer with a read length configuration of 2 × 150 bp.

### Quality control and read mapping

Raw paired-end reads were subjected to trimming and quality control using Fastp [23] with default parameters. Subsequently, the clean reads were individually aligned to a reference genome in orientation mode using HISAT2 software [24]. The mapped reads from each sample were then assembled using StringTie [25] with a reference-based approach.

### Differential expression analysis and functional enrichment

To identify differentially expressed genes (DEGs) between the *L. sphaericus* exposure group (AdLs) and the control group (AdCtrl), the expression level of each transcript was calculated using the transcripts per million reads (TPM) approach. The quantification of gene abundance was performed using RSEM (http://deweylab.biostat.wisc.edu/rsem/) [26]. The DEGseq [27] tool was utilized for differential expression analysis. DEGs with |log_2_ (foldchange)| ≧ 1 or a false discovery rate (FDR) ≤ 0.001 were considered significantly differentially expressed. Subsequently, a functional enrichment analysis was performed using Gene Ontology (GO) and Kyoto Encyclopedia of Genes and Genomes (KEGG) pathways to identify significantly enriched DEGs in GO terms and metabolic pathways with an adjusted *P*-value ≤ 0.05 compared to the entire transcriptome background. GO functional enrichment and KEGG pathway analysis were conducted using Goatools (https://github.com/ tanghaibao/Goatools) and KOBAS (http://kobas.cbi.pku.edu.cn/ home.do) [28], respectively. The functional implications of mRNA alterations were characterized using RNA-seq, and a Gene Set Enrichment Analysis (GSEA) was performed utilizing the GSEA software.

### Real-time quantitative PCR

Real-time quantitative PCR (qPCR) analysis was carried out to assess the transcriptional activity of crucial fertility-associated genes in *An. dirus*, including *vitellogenin* (Ad*Vg*), *target of rapamycin* (Ad*TOR*), *Sphingomyelin phosphodiesterase* (Ad*SMPD*), and *Niemann-Pick C2* (Ad*NPC2*) genes. The internal control for normalization was the *An. Dirus*' conserved S7 gene. Each 20 μl qPCR reaction comprised 0.8 μl of each primer at a concentration of 10 μM, 10 μl KAPA SYBR® FAST qPCR Kit Master Mix 2 × Universal (KAPA Biosystems, USA), 7.9 μl distilled water, and 0.5 μl synthesized cDNA. The qPCR process was executed on a Bio-Rad CFX96 Touch™ real-time PCR system with a 96-well reaction plate. The thermal cycling conditions for SYBR Green-based quantification involved an initial denaturation at 95 °C for 3 min and then 40 cycles of 95 °C for 3 s and 60 °C for 30 s. Subsequently, a melt curve analysis was conducted, gradually increasing the temperature from 65 to 95 °C at 0.5 °C increments every 5 s. Relative gene expression levels, normalized against ribosomal S7 RNA, were calculated using the 2^−ΔΔCT^ approach. The primers used are listed in Table S1 (see Additional file 1).

### Western blot

Proteins were extracted from 20 engorged mosquitoes per group using Western Blotting and Immunoprecipitation Cell Lysate kit (Beyotime, Shanghai, China), strictly following the manufacturer's guidelines for protein isolation. Subsequently, protein extracts were electrophoresed through 10% sodium dodecyl sulfate–polyacrylamide gels and transferred onto PVDF membranes. The membranes were blocked with 5% non-fat milk for 1 h, followed by overnight incubation with primary antibodies (diluted 1:1000) targeting pivotal fertility-related mosquito proteins, including Vg, AKT, TOR, phosphorylated AKT (p-AKT), and phosphorylated TOR (p-TOR), which serve as markers for TOR activity [29, 30]. Simultaneously, β-actin was used as a control protein and incubated at 4 °C. After three consecutive washes in TBST for 10 min each time, the membranes were treated with a secondary anti-rabbit antibody at room temperature for 1 h. Subsequently, the membranes underwent another round of washing before being imaged using the ChemiDOC^TM^MP Imaging System (BIO-RAD). The signal intensity was quantitatively assessed using ImageJ software.

### Statistical analysis

All statistical analyses were performed using IBM SPSS Statistic version 19.0 (SPSS Corp., Armonk, NY, USA). The Chi-square test [31] was applied to compare oviposition rates. Student’s t-test was employed to compare the control and experimental groups, while the Mann-Whitney *U*-test was used for non-normal distribution data in comparing egg counts between the experimental and control groups. One-way analysis of variance (ANOVA) was utilized to analyze differences among three or more groups. Statistical significance was set at *P* < 0.05.

## Results

### Presence of *Lysinibacillus sphaericus* in surviving adult mosquitoes post-sublethal dose exposure

**Fig. 1:**
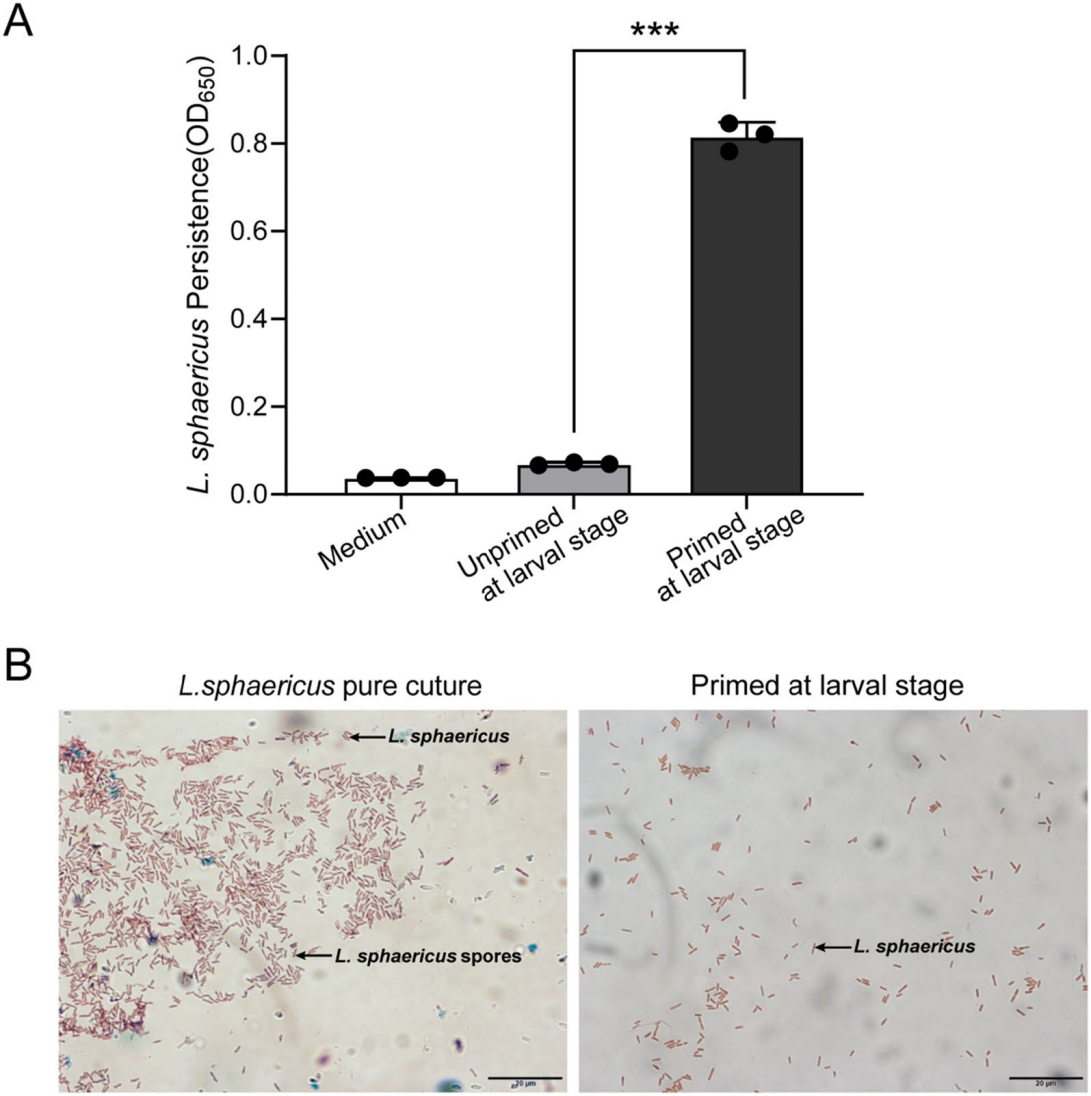
Evaluation of *Lysinibacillus sphaericus* persistence in adult female *Anopheles dirus* following larval exposure. **A** Persistence of *L. sphaericus* is indicated by the turbidity intensity of bacterial cultures in LB medium, quantified by measuring the optical density at 650 nm (OD_650_). ***Significant difference in OD_650_ values between unprimed (UPr) and primed (Pr) female mosquitoes at the larval stage. Each group consisted of 20 females. **B** Microscopic view of spore staining. The scale bar represents 20 μm

Our previous research demonstrated that exposure to *L. sphaericus* during the larval stage of *An. dirus* significantly diminished adult mosquito oviposition while increasing egg retention in their bodies [15]. To further investigate this mechanism, we conducted quantitative experiments on bacterial concentrations and staining assays to detect the persistence of *L. sphaericus* in surviving adults. Our results revealed a significantly higher optical density at 650 nm (OD_650_) for bacterial concentrations in the primed larval stage (Pr) group compared to the unprimed larval stage (UPr) group (Fig. 1A), indicating a greater abundance of bacteria in the Pr group (ANOVA, *F*_(2, 6)_ = 278.0, *P* < 0.0001). Additionally, spore staining revealed a similar morphology between bacteria isolated from the Pr group and the pure *L. sphaericus* strain, unlike in the UPr group (Fig. 1B). These findings suggest that *L. sphaericus* may exhibit long-term colonization in adult mosquitoes when *An. dirus* larvae are exposed to *L. sphaericus*.

### Effect of direct feeding of *L. sphaericus* on oviposition in adult *An. dirus*

**Fig. 2:**
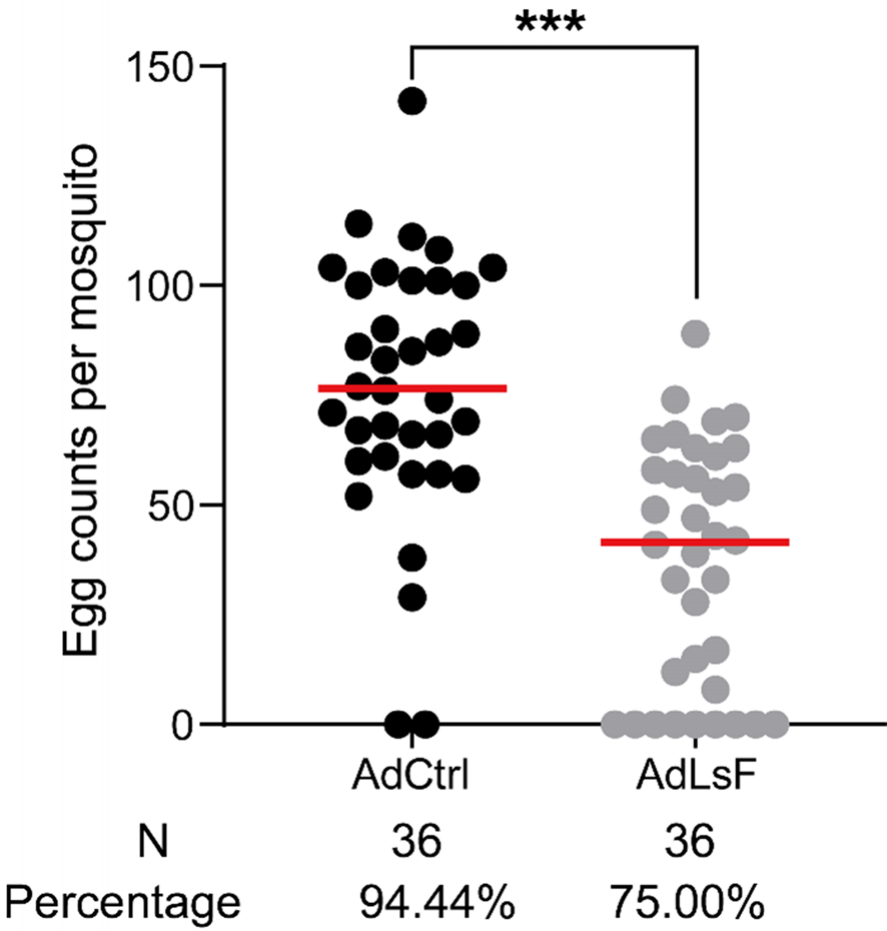
Egg counts comparison between control and AdLsF mosquitoes. The figure shows a significant reduction in the number of eggs laid by females subjected to direct feeding of *Lysinibacillus sphaericus* (AdLsF) compared to the control (AdCtrl) group. ***Statistically significant difference in egg counts between the control and AdLsF adults (*P* < 0.001)

To assess the effect of *L. sphaericus* on the oviposition ability of adult *An. dirus*, newly emerged mosquitoes were allowed to feed on a sucrose solution containing *L. sphaericus* for at least 3 consecutive days. Consistent with our previous findings, the number of eggs laid by females subjected to direct *L. sphaericus* feeding (AdLsF) was significantly reduced compared to the control group (Mann-Whitney U-test, *U* = 199.5, *Z* = − 5.061, *P* < 0.0001). Furthermore, the oviposition rate among AdLsF adults, at 75.00%, was markedly lower than the control group’s rate of 94.44% (Chi-square test, *χ*^*2*^ = 5.258, df = 1, *P* = 0.022 < 0.05) (Fig. 2). These results indicate that the presence of *L. sphaericus* in adult mosquitoes significantly hinders the oviposition capability of female *An. dirus*.

### Transcriptomic analysis of adult *An*. *dirus* with larval exposure to *L*. *sphaericus*

To explore the potential molecular mechanisms underlying the inhibition of fecundity in surviving adult *An. dirus* mosquitoes exposed to *L. sphaericus* during the larval stage, we focused on transcriptional changes occurring 24 h after blood feeding. This approach allowed us to compare gene expression profiles between the control and *L. sphaericus*-exposed groups, providing insights into the biological pathways affected by *L. sphaericus* exposure. The six samples yielded a total of 3.03 × 10^8^ raw reads (RR). Further details on the quality assessment and sequencing metrics are provided in Table S2 (see Additional file 2). A total of 11,411 genes were obtained through sequencing, and gene expression levels were normalized using TPM (transcripts per million) values. The distribution of expression levels in each sample is illustrated in Fig. S1 (see Additional file 3).

**Fig. 3:**
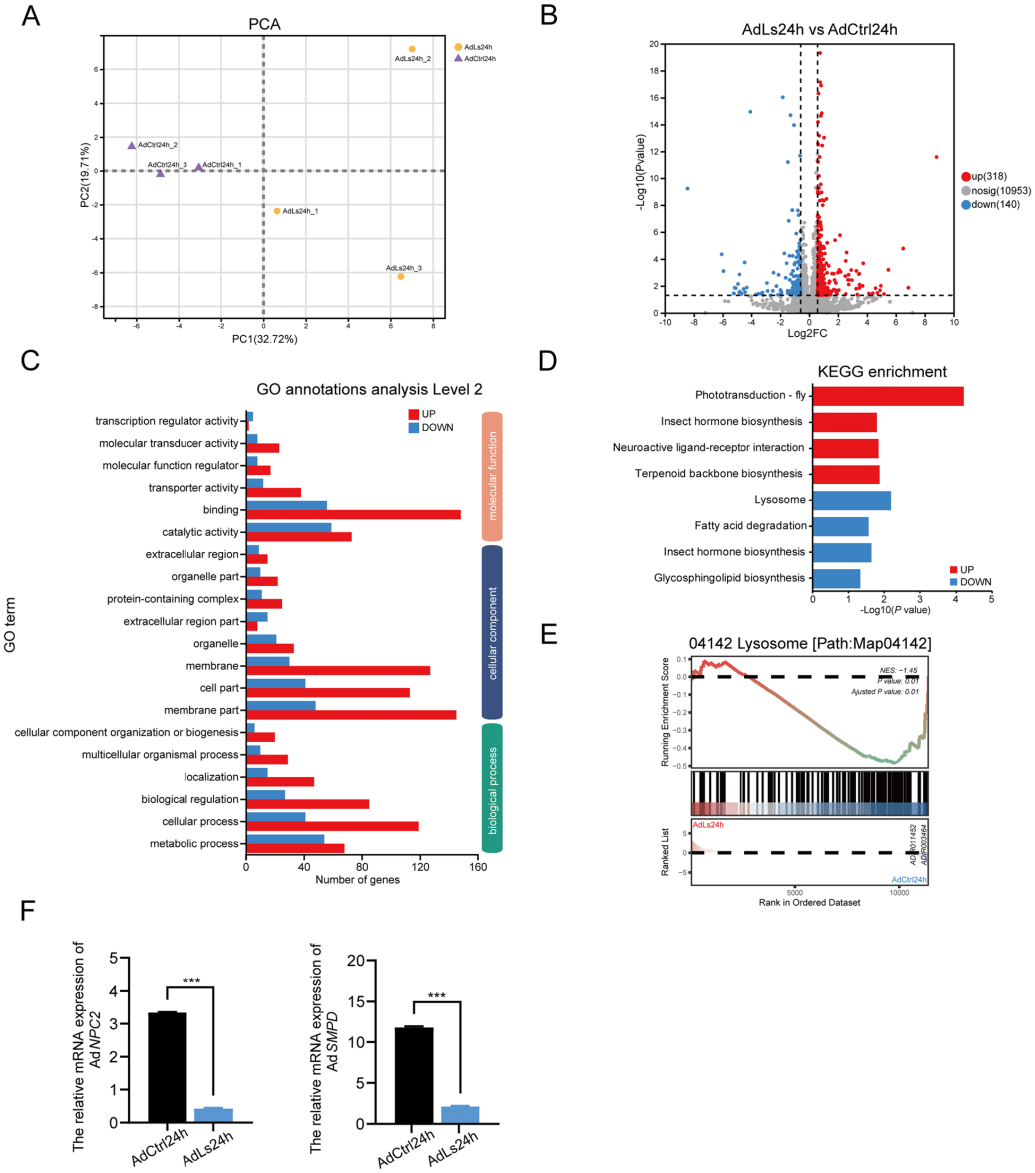
Transcriptome sequencing analysis of adult *Anopheles dirus* with larval exposure to *Lysinibacillus sphaericus*. AdLs24h: Adult *An. dirus* exposure to *L. sphaericus* during larval stage, sampled 24 h post-blood meal (PBM); AdCtrl24h: Unexposed adult *An. dirus*, sampled 24 h PBM. **A** Principal Component Analysis (PCA) illustrates sample similarity, with proximity indicating higher similarity. The horizontal axis (PC1) and vertical axis (PC2) represent the principal components' contributions to sample discrimination. **B** Volcano plot showing differentially expressed genes between the AdLs24h and AdCtrl24h groups. **C** Gene Ontology (GO) functional enrichment analysis of differentially expressed genes. **D** KEGG Pathway Enrichment Plot indicating significantly enriched pathways in the AdLs24h group, with blue for downregulated and red for upregulated genes. **E** Gene Set Enrichment Analysis (GSEA) for the lysosome pathway, demonstrating significant enrichment in the AdLs24h group (NES = 1.45, *P*-value = 0.01, adjusted *P*-value = 0.01). **F** mRNA expression levels of Ad*NPC2* and Ad*SMPD* at 24 h PBM. ***Significant differences (*P* < 0.001) between AdLs24h and AdCtrl24h groups

By comparing gene expression profiles, we observed that the transcriptional patterns between the two groups were markedly distinct (Fig. 3A). This analysis led to the identification of 458 differentially expressed genes. Specifically, 318 genes exhibited upregulation, while 140 genes showed downregulation following larval exposure to *L. sphaericus*, with the criteria set as |log_2_ (fold change)|≥ 0.585 and *P*-value < 0.05 (Fig. 3B, Additional file 4: Fig. S2). Furthermore, the functional enrichment analysis of these differentially expressed genes was primarily centered on metabolic and cellular processes and biological regulation (Fig. 3C). Significantly upregulated genes in the *L. sphaericus* exposure group were enriched in processes related to phototransduction (map04745), neuroactive ligand-receptor interactions (map04080), and terpenoid backbone biosynthesis (map00900). Conversely, significantly downregulated genes were enriched in processes associated with lysosomes (map04142), fatty acid degradation (map00071), and glycosphingolipid biosynthesis (map00603). Notably, both up- and downregulated gene sets were enriched in insect hormone biosynthesis (map00981) (Fig. 3D). The GSEA analysis revealed significant changes in the lysosome pathway (Path Map04142) in response to larval exposure to *L. sphaericus*. The Normalized Enrichment Score (NES) for this pathway was 1.45, with a *P*-value of 0.01 and an adjusted *P*-value also at 0.01, indicating a statistically significant enrichment (Fig. 3E). This suggests that larval exposure to *L. sphaericus* has a notable impact on lysosomal function. Furthermore, the qPCR results confirmed the suppression of lysosomal function-related genes in the *L. sphaericus* exposure group compared to the control. Specifically, the mRNA expression levels of *NPC2* and *SMPD* were significantly reduced in the *L. sphaericus* exposure group, as shown in Fig. 3F. The *P*-values for *NPC2* and *SMPD* were both < 0.001, indicating a highly significant downregulation. Specifically, the t-test results for *NPC2* showed *t*_(4)_ = 88.81, *P* < 0.001, and for *SMPD*, *t*_(4)_ = 55.44, *P* < 0.001. These findings are consistent with the GSEA results and further support the conclusion that larval exposure to *L. sphaericus* affects lysosomal function.

### Role of Vg and TOR signaling pathway in *L*. *sphaericus*-induced inhibition of oviposition in *An*. *dirus*

**Fig. 4:**
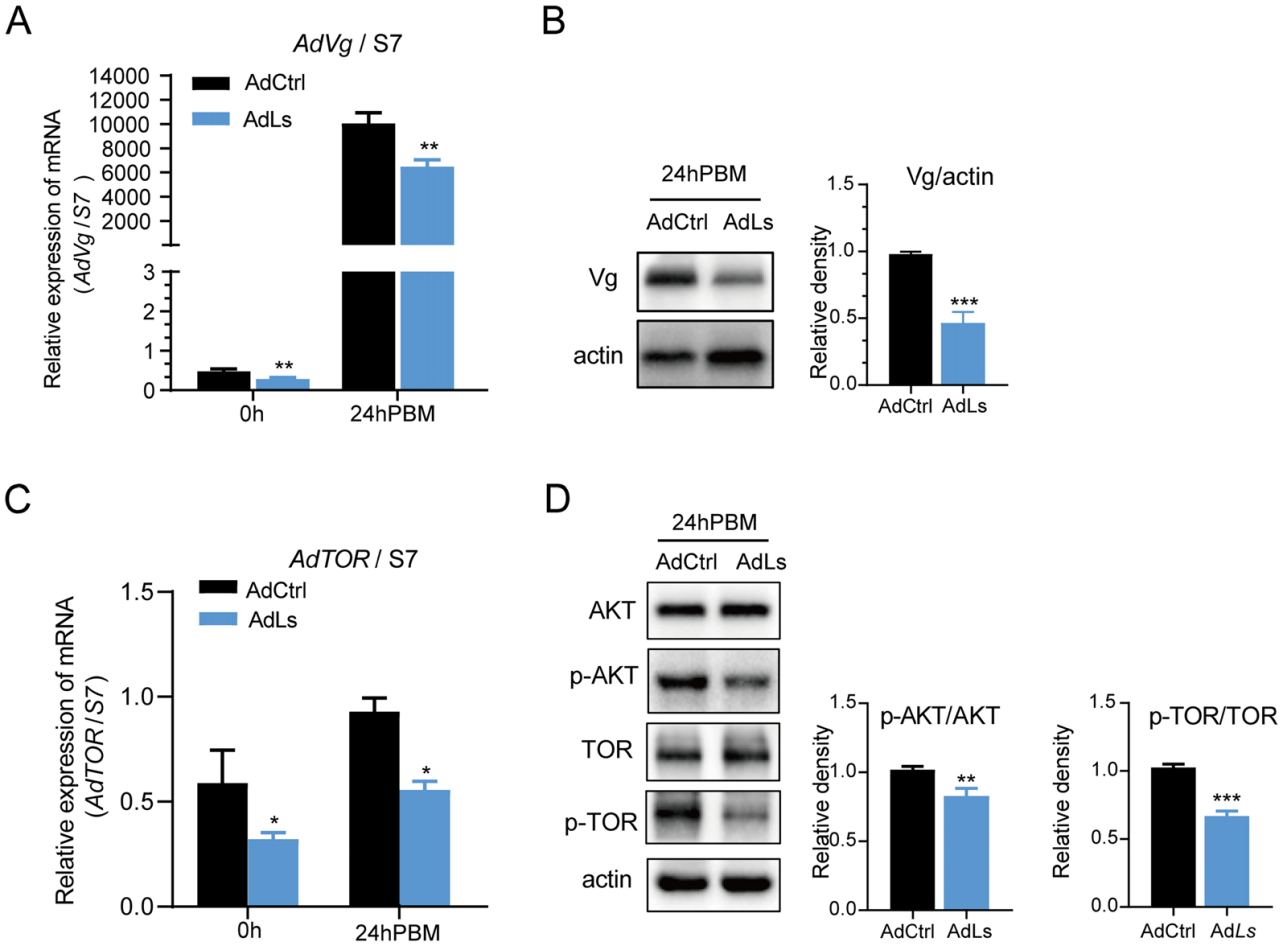
Effects of *Lysinibacillus sphaericus* exposure on gene and protein expression levels in *Anopheles dirus*. AdLs: Adult *An. dirus* exposure to *L. sphaericus* during larval stage; AdCtrl: unexposed adult *An. dirus*. **A** Real-time quantitative PCR analysis showing Ad*Vg* mRNA levels in female mosquitoes at 0 h and 24 h post-blood meal (PBM). **B** Western blot analysis displaying Vg protein levels in female mosquitoes at 24 h PBM. **C** Real-time quantitative PCR analysis of Ad*TOR* mRNA levels in female mosquitoes at 0 h and 24 h PBM. **D** Western blot analysis of AKT and TOR phosphorylation levels in female mosquitoes at 24 h PBM. Asterisks denote statistical significance: ***P < 0.001, **P < 0.01, *P < 0.05

We initially evaluated the transcription and protein levels of vitellogenin (Vg) in surviving female adults treated with sublethal doses of *L. sphaericus* during the larval stage using qPCR and Western blotting techniques. Exposure to *L. sphaericus* significantly inhibited Ad*Vg* expression in female mosquitoes, as shown in Fig. 4A and B. Specifically, qPCR revealed a significant decrease in Ad*Vg* mRNA levels in *L. sphaericus*-exposed group (AdLs) mosquitoes at both 0 h (t-test, *t*_(4)_ = 4.85, *P* = 0.008) and 24 h (*t*_(4)_ = 5.93, *P* = 0.004) post-blood meal (PBM). Correspondingly, Western blot analysis showed a suppressed level of Vg protein at 24 h PBM in surviving female mosquitoes (*t*_(4)_ = 10.54, *P* = 0.0005).

To further investigate the potential mechanisms underlying the downregulation of Ad*Vg* expression, we examined the activation of the TOR signaling pathway, which is known to regulate vitellogenesis. The results, depicted in Fig. 4C and D, indicated that the activation of the TOR signaling pathway was also suppressed in *L. sphaericus*-exposed (AdLs) mosquitoes. Real-time quantitative PCR analysis showed a significant decrease in Ad*TOR* mRNA levels at both 0 h (*t*_(4)_ = 2.905, *P* = 0.04) and 24 h (*t*_(4)_ = 8.428, *P* = 0.01) PBM. Western blot analysis further confirmed the inhibition of AKT and TOR phosphorylation levels in *L. sphaericus*-exposed (AdLs) adults, with *P*-values of 0.0053 (*t*_(4)_ = 5.512) and 0.0002 (*t*_(4)_ = 13.93), respectively. These findings demonstrate that larval exposure to *L. sphaericus* leads to significant downregulation of vitellogenin expression and inhibition of the TOR signaling pathway, which are likely contributing factors to the observed reduction in oviposition ability.

**Fig. 5:**
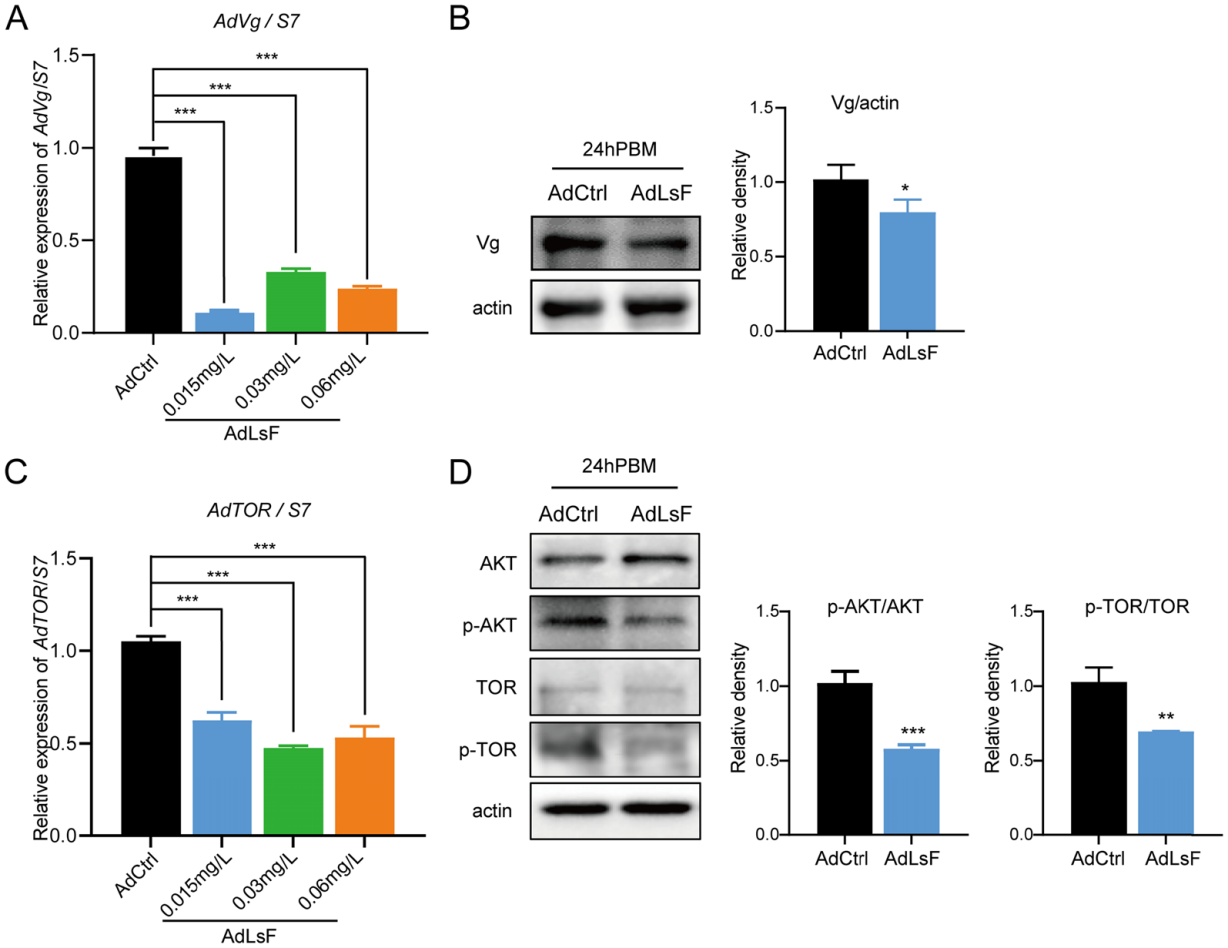
Direct ingestion of *Lysinibacillus sphaericus* affects Ad*Vg* expression and TOR signaling pathway activation in adult *Anopheles dirus*. AdLsF: Adult *An. dirus* mosquitoes subjected to direct feeding of *L. sphaericus*; AdCtrl: normal adult *An. dirus* mosquitoes (unexposed to *L. sphaericus*). **A** mRNA levels of Ad*Vg* in female mosquitoes at 24 h post-blood meal (PBM) following exposure to varying concentrations of *L. sphaericus* (0.015 mg/l, 0.03 mg/l, 0.06 mg/l; *P* < 0.001 for all). **B** Protein levels of Vg in female mosquitoes at 24 h PBM after direct feeding of *L. sphaericus* at 0.015 mg/l (*P* = 0.0421). **C** mRNA levels of Ad*TOR* in female mosquitoes at 24 h PBM following exposure to varying concentrations of *L. sphaericus* (0.015 mg/l, 0.03 mg/l, 0.06 mg/l; *P* < 0.001 for all). **D** Phosphorylation levels of AKT and TOR in female *An. dirus* after direct feeding of *L. sphaericus* (0.015 mg/l) (*P* = 0.0007 and 0.0041, respectively). Asterisks denote statistical significance: ***P < 0.001, **P < 0.01, *P < 0.05

To further substantiate the impact of *L. sphaericus* on the ovipositional capacity of *An. dirus*, we incorporated different concentrations of *L. sphaericus* into a sucrose solution for direct feeding of adult *Anopheles* mosquitoes and subsequently evaluated Ad*Vg* expression levels. The findings indicated that direct feeding of *L. sphaericus* (AdLsF) significantly reduced Ad*Vg* mRNA levels in adult mosquitoes fed with sucrose solutions containing *L. sphaericus* at concentrations of 0.015 mg/l (ANOVA, *F*_(3, 8)_ = 229.457, *P* < 0.001), 0.03 mg/l (*F*_(3, 8)_ = 229.457, *P* < 0.001), and 0.06 mg/l (*F*_(3, 8)_ = 229.457, *P* < 0.001) (Fig. 5A). Correspondingly, Vg protein levels were decreased at 24 h post-blood meal (PBM) in female mosquitoes fed with the lowest concentration of *L. sphaericus* (0.015 mg/l, *t*_(4)_ = 2.947, *P* = 0.0421) (Fig. 5B). Additionally, the activation of the TOR signaling pathway was assessed, revealing significant inhibition of Ad*TOR* mRNA levels upon direct feeding of *L. sphaericus* at concentrations of 0.015 mg/l (ANOVA, *F*_(3, 8)_ = 42.956, *P* < 0.001), 0.03 mg/l (*F*_(3, 8)_ = 42.956, *P* < 0.001), and 0.06 mg/l (*F*_(3, 8)_ = 42.956, *P* < 0.001) (Fig. 5C). Furthermore, phosphorylation levels of AKT and TOR were notably decreased (Fig. 5D), with *P*-values of 0.0007 (*t*_(4)_ = 9.605) and 0.0041 (*t*_(4)_ = 5.920), respectively, indicating a statistically significant inhibition. These results demonstrate that ingestion of *L. sphaericus* at various concentrations leads to a downregulation of *Vg* expression and a reduction in TOR signaling pathway activation in adult *An. dirus* mosquitoes.

## Discussion

*Lysinibacillus sphaericus* is a widely distributed gram-positive bacterium known for its efficacy as a larvicidal agent against mosquito larvae [32]. The toxicity of *L. sphaericus* is primarily mediated through the production of toxic proteins, such as BinA and BinB [33, 34], which target midgut receptors in mosquito larvae, leading to cell destruction and larval mortality [35, 36]. *Lysinibacillus sphaericus* is a pivotal agent in mosquito control due to its high specificity and toxicity towards insect larvae, particularly mosquitoes, while remaining harmless to other organisms and having a negligible environmental impact [37]. However, the effectiveness of *L. sphaericus* in field applications is influenced by multiple factors, including mosquito species, feeding behaviors, environmental conditions, and formulation properties (e.g. type, toxin concentration, delivery, and sedimentation rate) [37]. These factors highlight the complexity of deploying *L. sphaericus* in mosquito control programs and underscore the importance of optimizing the use of biological insecticides.

Our previous study showed that larval exposure to *L. sphaericus* significantly suppressed the fecundity of surviving *An. dirus* adult female mosquitoes [15], although the underlying mechanism remains unclear. In this study, we found that *L. sphaericus* can persist in adult mosquitoes that survived larval exposure, impeding their oviposition ability. This finding aligns with reports that sublethal doses of *L. sphaericus* can influence the development of *Wuchereria bancrofti* parasites in *Culex* mosquitoes, highlighting the broader ecological implications of *L. sphaericus* exposure [38]. These insights enhance our understanding of the multifaceted effects of *L. sphaericus* on mosquito populations and guide the development of more effective and sustainable strategies for integrated mosquito management.

To further elucidate the mechanisms underlying the inhibitory effects of *L. sphaericus* on the oviposition ability of *An. dirus* mosquitoes, we conducted transcriptome sequencing analysis. Our results revealed differential expression of 458 genes following *L. sphaericus* treatment, with significant associations in pathways related to phototransduction, insect hormone biosynthesis, lysosomal function, fatty acid degradation, and glycosphingolipid biosynthesis. These findings suggest that *L. sphaericus* may disrupt key physiological processes in mosquitoes, ultimately affecting their reproductive fitness.

Female mosquitoes typically initiate host-seeking behavior to obtain blood meals and oviposit approximately 72 h after emergence. Nutritional signals from blood meals trigger the activation of the AKT/TOR signal pathway, which promotes the sequential synthesis of ecdysteroids from cholesterol in both the endoplasmic reticulum and mitochondria of follicle cells [39]. This active hormone induces the expression of vitellogenin (*Vg*) in the fat body, which is then release into the hemolymph, taken up by oocytes, and packaged into yolk granules [40, 41]. The Niemann-Pick C2 protein (NPC2) and Sphingomyelin phosphodiesterase (SMPD) are key lysosomal proteins involved in cholesterol transport and sphingomyelin metabolism, respectively [42, 43]. In this study, we found that *L. sphaericus* significantly inhibited the expression of *NPC2* and *SMPD*, potentially lead to cholesterol accumulation in lysosomes, suppression of ecdysteroid biosynthesis, and reduced *Vg* expression levels. Additionally, our analysis of transcription and protein phosphorylation levels revealed that *L. sphaericus* treatment inhibited the AKT/TOR signaling pathway, further suppressing ecdysteroid synthesis and Vg protein production. These molecular changes ultimately impair the oviposition ability of *An. dirus* mosquitoes.

However, several intriguing questions remain unanswered. For instance, the precise mechanisms by which *L. sphaericus* inhibits the expression of *NPC2* and *SMPD* are still unclear. Overall, our findings demonstrate that *L. sphaericus* treatment can downregulate *Vg* expression through two distinct pathways: inhibition of lysosomal function and suppression of the TOR signaling pathway. These interventions ultimately lead to a significant suppression of oviposition capacity in *An*. *dirus*. Despite these promising results, the use of sublethal doses of *L. sphaericus* is not recommended, as they may contribute to the development of resistance [15]. Future studies should focus on elucidating the detailed molecular mechanisms underlying these effects and exploring strategies to optimize *L. sphaericus* application while minimizing the risk of resistance development.

## Conclusions

This study investigated the potential mechanisms underlying the inhibitory effects of sublethal doses of *Lysinibacillus sphaericus* (*L. sphaericus*) on the reproductive capacity of *An. dirus*. Our results demonstrated that *L. sphaericus* persist in surviving adult *An*. *dirus* mosquitoes following larval exposure to sublethal dose, and direct feeding of *L. sphaericus* to adult females similarly impairs the oviposition ability. Transcriptome analysis revealed that sublethal doses of *L. sphaericus* significantly altered the transcriptional profiles of surviving mosquitoes, potentially disrupting key physiological processes. Specifically, *L. sphaericus* appears to inhibit lysosomal function and lipid metabolism, while significantly suppressing the Akt/TOR signaling pathway downregulating *Vg* expression in adult mosquitoes. Collectively, these findings provide novel insights into the host-pathogen interactions between *L. sphaericus* and mosquitoes and highlight a potential molecular strategy for controlling mosquito population density by modulating oviposition behavior.

## Supplementary Information

Additional file 1: Table S1. Primers used for qPCR.

Additional file 2: Table S2. Summary of quality control metric for transcriptome sequencing and alignment to the reference genome of *Anopheles dirus*.

Additional file 3: Fig. S1. Distribution of gene expression in different samples.

Additional file 4: Fig. S2. Heatmap of differentially expressed genes.

## Data Availability

RNA sequencing data are publicly available from the Gene Expression Omnibus (GEO) (accession code GSE272494).

## Abbreviations

*An. dirus*: *Anopheles dirus*
*L. sphaericus*: *Lysinibacillus sphaericus*
DEGs: Differentially expressed genes
TPM: Transcripts per million reads
GO: Gene Ontology
KEGG: Kyoto Encyclopedia of Genes and Genomes
GSEA: Gene Set Enrichment Analysis
Vg: Vitellogenin
TOR: Target of rapamycin
SMPD: Sphingomyelin phosphodiesterase
NPC2: Niemann–Pick C2 protein
S6K: S6 kinase
