# Supplementary material for: Lysinibacillus sphaericus exposure impedes Anopheles dirus’s oviposition via downregulating vitellogenin
Source: Parasit Vectors. 2025 Mar 21;18:111. doi: 10.1186/s13071-025-06745-8 (PMC11927181; doi:10.1186/s13071-025-06745-8)
Supplement: Supplementary file 1 — Additional file 1: Table S1. Primers used for qPCR. [file 13071_2025_6745_MOESM1_ESM.docx]

Additional file 1: Table S1. Primers used for qPCR.

| Gene name | Forward primer (5’-3’) | Reverse primer (5’-3’) |
| --- | --- | --- |
| **Ad*Vg*** | CACCATCCAGTCGGTGTCT | GTCGTAAGGCGTGATTTCGT |
| **Ad*TOR*** | ACGAGCGCGAACATCCTTAT | CACCACCGCCGGATAGTATT |
| **Ad*SMPD*** | GTCCGACTGCTTCAGCGTGTG | TTGAACTCGTCCTTGTGCGTGTG |
| **Ad*NPC2*** | ATGCGGCTGCTGGCTATTGTG | GGGACCTTCGGAACACTGATGC |
| **Ad*S7*** | CAACAACAAGAAGGCGATCA | GACGTGCTTACCGGAGAACT |
