# Supplementary material for: Lysinibacillus sphaericus exposure impedes Anopheles dirus’s oviposition via downregulating vitellogenin
Source: Parasit Vectors. 2025 Mar 21;18:111. doi: 10.1186/s13071-025-06745-8 (PMC11927181; doi:10.1186/s13071-025-06745-8)
Supplement: Supplementary file 2 — Additional file 2: Table S2. Summary of quality control metric for transcriptome sequencing and alignment to the reference genome of Anopheles dirus. [file 13071_2025_6745_MOESM2_ESM.docx]

Additional file 2: Table S2. Summary of Quality Control Metric for Transcriptome Sequencing and Alignment to the Reference Genome of *Anopheles dirus*

| Sample | Raw reads | Clean reads | Clean bases | Q20(%) | Q30(%) | GC content(%) | Total mapped | TM/CR |
| --- | --- | --- | --- | --- | --- | --- | --- | --- |
| AdLs24h_1 | 45595174 | 45279782 | 6749125277 | 98.09 | 94.30 | 51.64 | 37973221 | 83.86% |
| AdLs24h_2 | 49521918 | 49161992 | 7326421085 | 98.04 | 94.19 | 51.83 | 41564005 | 84.54% |
| AdLs24h_3 | 53487796 | 53102598 | 7893387000 | 98.09 | 94.28 | 50.87 | 44192035 | 83.22% |
| AdCtrl24h_1 | 51544390 | 51162576 | 7613087993 | 98.03 | 94.20 | 52.05 | 41782107 | 81.67% |
| AdCtrl24h_2 | 50570818 | 50236512 | 7498255644 | 98.10 | 94.32 | 52.65 | 42975853 | 85.55% |
| AdCtrl24h_3 | 52351714 | 52014850 | 7760599273 | 98.14 | 94.45 | 52.50 | 44909404 | 86.34% |

Note: **AdLs24h**: Adult *An. dirus* exposure to *L. sphaericus* during larval stage, sampled 24 h post-blood meal (PBM); **AdCtrl24h**: Unexposed adult *An. dirus*, sampled 24 h PBM; **Sample:** Designation of each sample analyzed; **Clean reads:** The total count of high-quality reads remaining after quality control procedures; **Clean bases:** The total quantity of high-quality sequence data obtained after quality control, determined by multiplying the number of clean reads by their average length; **Q20 (%) and Q30 (%):** Quality metrics for sequencing data post-quality control. Q20 and Q30 denote the proportion of bases with a quality score exceeding 20 (equivalent to an error rate of 1%) and 30 (equivalent to an error rate of 0.1%), respectively. Typically, a Q20 value above 85% and a Q30 value above 80% are considered acceptable; **GC content (%):** The proportion of guanine (G) and cytosine (C) bases in the high-quality sequence data; **Total mapped:** The number of clean reads successfully aligned or mapped to the reference genome, indicating the alignability of the sequencing data.
