# Supplementary material for: Lysinibacillus sphaericus exposure impedes Anopheles dirus’s oviposition via downregulating vitellogenin
Source: Parasit Vectors. 2025 Mar 21;18:111. doi: 10.1186/s13071-025-06745-8 (PMC11927181; doi:10.1186/s13071-025-06745-8)
Supplement: Supplementary file 3 — Additional file 3: Fig. S1. Distribution of gene expression in different samples. [file 13071_2025_6745_MOESM3_ESM.docx]

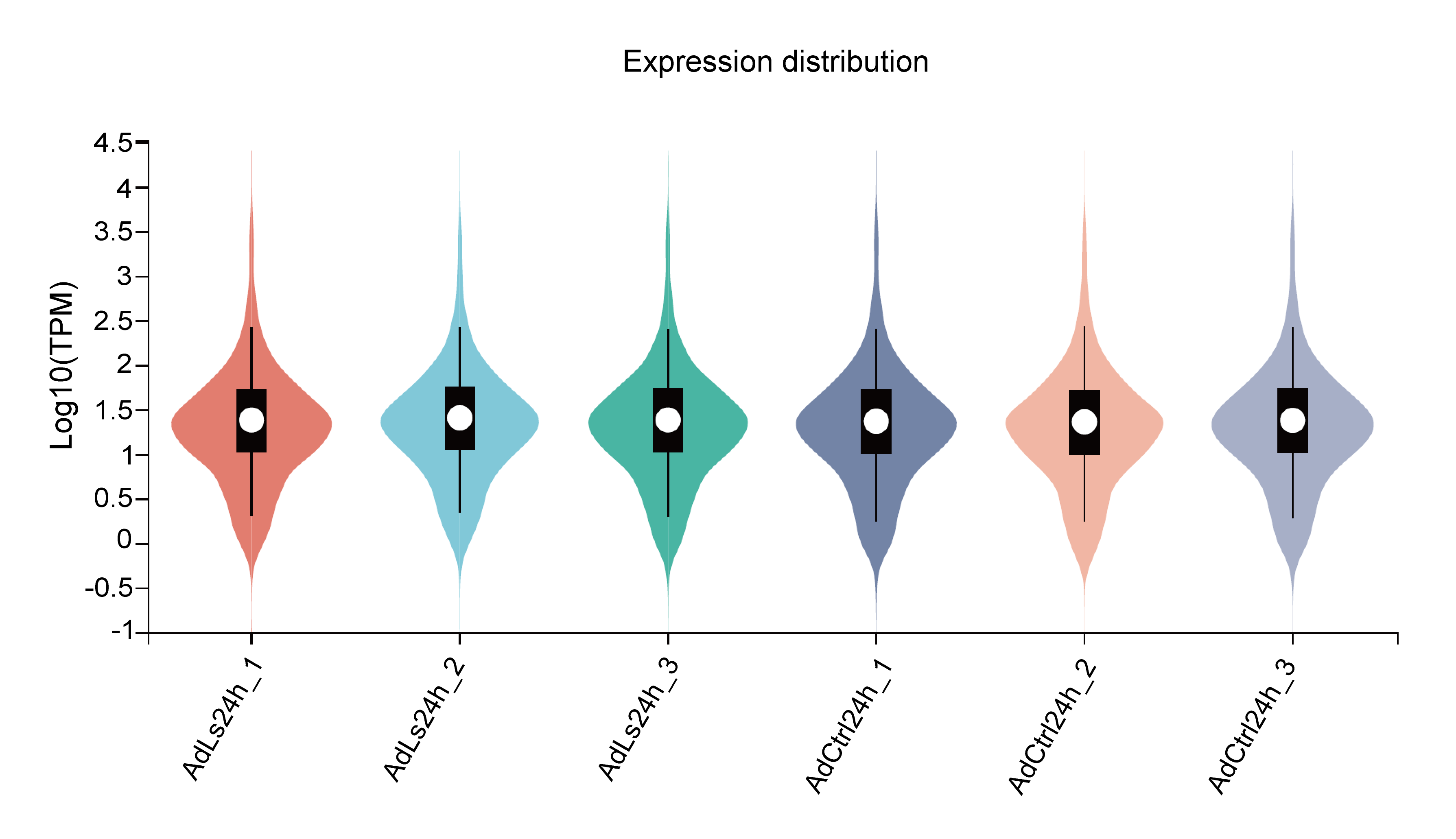


Additional file 3: **Fig. S1.** Distribution of gene expression in different samples. The x-axis represents the sample name, while the y-axis represents the expression quantity after log10 transformation. Each color corresponds to a specific sample, and the enlarged section indicates the region with the highest concentration of gene expression across all samples.
