# Supplementary material for: Lysinibacillus sphaericus exposure impedes Anopheles dirus’s oviposition via downregulating vitellogenin
Source: Parasit Vectors. 2025 Mar 21;18:111. doi: 10.1186/s13071-025-06745-8 (PMC11927181; doi:10.1186/s13071-025-06745-8)
Supplement: Supplementary file 4 — Additional file 4: Fig. S2. Heatmap of differentially expressed genes. [file 13071_2025_6745_MOESM4_ESM.docx]

**
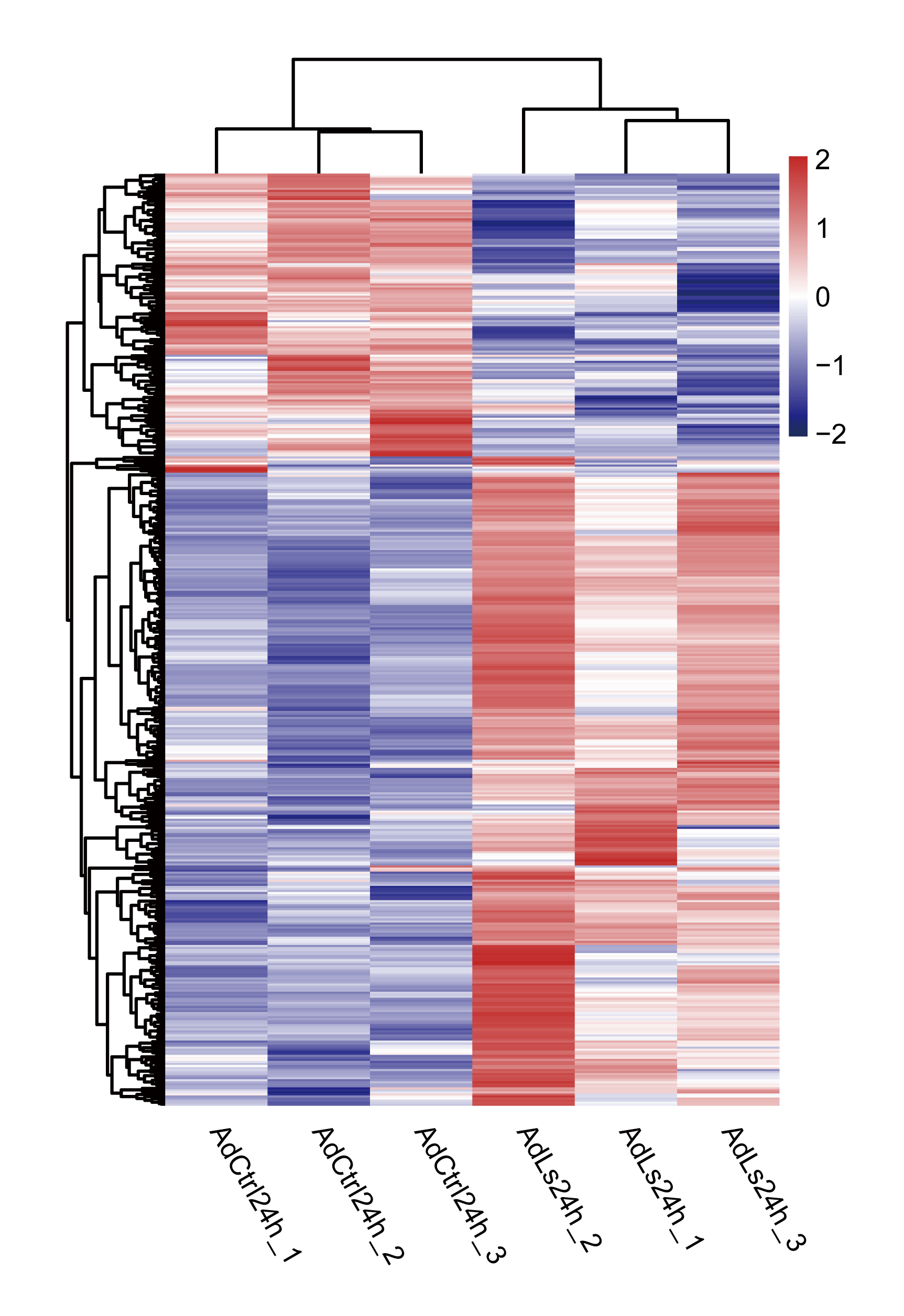
**

Additional file 4: **Fig. S2.** Heatmap of differentially expressed genes in AdLs24h compared to AdCtrl24h, with upregulated genes shown in red and downregulated genes in blue.
